# Tezepelumab in Chronic Rhinosinusitis with Nasal Polyps: Pathophysiology, Clinical Evidence, and Therapeutic Perspectives

**DOI:** 10.3390/medicina62071423

**Published:** 2026-07-22

**Authors:** Bayan Aigozhina, Rais Tulebaeyv, Talapbek Azhenov, Serik Dzhandayev, Nataliya Papulova, Rano Zhankina, Kalamkas Sagandykova

**Affiliations:** 1 Department of Otorhinolaryngology, Astana Medical University, Astana 010000, Kazakhstan; aigozhina.b@amu.kz (B.A.); tulebaev.r@amu.kz (R.T.); azhenov.t@amu.kz (T.A.); zhandayev.s@amu.kz (S.D.); papulova.n@amu.kz (N.P.); zhankina.r@amu.kz (R.Z.); 2 “University Medical Center” Corporate Fund, School of Medicine, Astana 010000, Kazakhstan

**Keywords:** chronic rhinosinusitis with nasal polyps, CRSwNP, tezepelumab, TSLP, epithelial alarmins, biological therapy, type 2 inflammation

## Abstract

*Background and Objectives:* Chronic rhinosinusitis with nasal polyps (CRSwNP) is a heterogeneous inflammatory disease of the nasal and paranasal sinus mucosa, associated with significant impairment in quality of life, frequent postoperative recurrence, and repeated need for systemic glucocorticosteroid therapy. Despite the availability of biologics targeting IL-4/IL-13, IL-5, and IgE, a subset of patients shows incomplete or insufficient clinical response. In this context, upstream targeting of epithelial alarmins, particularly thymic stromal lymphopoietin (TSLP), has emerged as a potential therapeutic strategy. To critically review current evidence on the role of TSLP in CRSwNP and to evaluate available data on the mechanism of action, clinical efficacy, and therapeutic potential of tezepelumab in severe and recurrent disease. *Materials and Methods*: A narrative review was conducted using PubMed, Scopus, and Web of Science. Studies published between 2016 and 2026 were included, comprising experimental research, phase II–III clinical trials, systematic reviews, and international guidelines. *Results*: TSLP functions as an epithelial alarmin that initiates and amplifies type 2 inflammation via dendritic cell activation, Th2 polarization, and activation of type 2 innate lymphoid cells (ILC2). Data suggests that tezepelumab, a monoclonal antibody targeting TSLP, may reduce inflammation and regulate the immune system. Evidence from asthma populations and relevant CRSwNP subgroups indicates potential improvements in nasal polyp score, congestion, olfactory function and quality of life. It is our understanding that the safety profile appears comparable to placebo, with no new safety concerns having been identified in long-term studies. *Conclusions*: Tezepelumab is a promising biologic that targets inflammation in CRSwNP. It may benefit severe, recurrent, treatment-resistant disease by modulating immune pathways. However, evidence is indirect and limited, and more trials are needed to define its efficacy, identify biomarkers, and clarify its role in treatment algorithms.

## 1. Introduction

Chronic rhinosinusitis with nasal polyps (CRSwNP) is a heterogeneous inflammatory disorder of the sinonasal mucosa characterised by persistent inflammation, recurrent symptoms, and substantial impairment of quality of life [1,2,3,4,5]. The disease affects approximately 2–4% of the adult population and commonly presents with nasal obstruction, rhinorrhoea, facial pressure, and impaired olfaction ranging from hyposmia to complete anosmia [1,6,7]. Beyond its local manifestations, CRSwNP imposes a considerable disease burden, with an impact on quality of life comparable to that observed in other severe chronic respiratory disorders [8,9,10].

Current management strategies for severe CRSwNP rely primarily on topical and systemic glucocorticosteroids, with functional endoscopic sinus surgery (FESS) reserved for patients with inadequate disease control despite optimal medical therapy [1,3]. Although these approaches remain essential components of disease management, they fail to provide sustained long-term control in a substantial proportion of patients [1,3,11,12]. Although endoscopic sinus surgery remains an effective treatment option for patients with severe CRSwNP, postoperative disease recurrence continues to represent a significant clinical challenge. According to the meta-analysis by Loftus et al., revision surgery rates following primary endoscopic sinus surgery range from approximately 14% to 24%, depending on patient characteristics, duration of follow-up, and underlying inflammatory endotypes [5]. The risk of recurrence is particularly high in patients with severe eosinophilic inflammation and comorbid asthma, highlighting the need for more effective long-term disease control strategies [12,13].

Furthermore, prolonged systemic corticosteroid exposure is limited by cumulative adverse effects, including metabolic complications, osteoporosis, and secondary adrenal insufficiency [1,14]. The EPOS 2020 consensus document highlights the continuing unmet need for novel therapeutic strategies capable of achieving durable disease control in patients with severe CRSwNP despite established treatment modalities [1].

In recent years, advances in the understanding of CRSwNP pathogenesis have led to the recognition of distinct inflammatory endotypes, fundamentally transforming therapeutic approaches and enabling the development of targeted biological therapies [1,15,16,17]. In Western populations, CRSwNP is predominantly characterised by type 2 inflammation, involving activation of the Th2 immune pathway, eosinophilic infiltration of the sinonasal mucosa, and increased expression of type 2 cytokines, including IL-4, IL-5, and IL-13 [1,18,19]. This improved understanding of disease endotypes has facilitated the integration of biologic therapies into the management of severe CRSwNP, with currently approved agents targeting key type 2 inflammatory pathways, including IL-4/IL-13 signalling (dupilumab), IL-5-mediated eosinophilic inflammation (mepolizumab and reslizumab), and IgE-dependent mechanisms (omalizumab) [1,15,20,21,22].

Despite the substantial clinical benefits achieved with currently available biologics, a subset of patients continues to demonstrate persistent inflammatory activity or an incomplete therapeutic response [22]. These limitations suggest that additional regulatory mechanisms located upstream within the inflammatory cascade may contribute to disease persistence and represent potential therapeutic targets.

Consequently, increasing attention has been directed towards epithelial alarmins, including thymic stromal lymphopoietin (TSLP), IL-25, and IL-33, which function as early regulators of type 2 inflammatory responses [23]. In CRSwNP, accumulating evidence indicates that TSLP, together with IL-25 and IL-33, plays a central role in initiating and amplifying type 2 inflammation [23,24,25,26]. A variety of infectious, allergic, and environmental stimuli can induce TSLP expression in epithelial cells, thereby contributing to the activation and perpetuation of inflammatory pathways within the sinonasal mucosa [27,28]. TSLP promotes dendritic cell activation, drives Th2 cell polarisation, and enhances type 2 innate lymphoid cell (ILC2) responses, thereby contributing to persistent eosinophilic inflammation and tissue remodelling in CRSwNP [26,27,29].

Tezepelumab (Amgen Inc., Thousand Oaks, CA, USA; AstraZeneca, Cambridge, UK), a fully human monoclonal antibody targeting TSLP, represents the first clinically available anti-TSLP biologic therapy designed to inhibit inflammatory signalling at an upstream level [30,31]. Unlike conventional biologics that target individual cytokines, TSLP inhibition has the potential to modulate both innate and adaptive immune responses, thereby affecting a broader spectrum of inflammatory endotypes, including mixed and partially non-type 2 phenotypes [26,32,33].

Despite significant advances in biological therapy for CRSwNP, the clinical role of tezepelumab in this disease remains to be fully defined. Therefore, this review aims to analyse the pathophysiological mechanisms underlying TSLP-mediated inflammation, evaluate the clinical efficacy and safety profile of tezepelumab, and discuss its potential role within personalised treatment strategies for patients with severe CRSwNP.

## 2. Materials and Methods

This narrative review evaluates the role of thymic stromal lymphopoietin (TSLP) in the pathogenesis of chronic rhinosinusitis with nasal polyps (CRSwNP) and examines the therapeutic potential of tezepelumab as a targeted biological therapy.

A comprehensive literature search was conducted in the PubMed, Scopus, and Web of Science databases. The review included publications from 2016 to 2026, encompassing basic, translational, and clinical studies, as well as international guidelines on the diagnosis and management of CRSwNP.

The search strategy included the following keywords and their combinations: “chronic rhinosinusitis with nasal polyps”, “CRSwNP”, “tezepelumab”, “thymic stromal lymphopoietin”, “TSLP”, “epithelial alarmins”, “type 2 inflammation”, and “biologic therapy”. Boolean operators (AND/OR) were applied to refine and optimise the search strategy.

Eligible studies included: (1) original clinical trials, including phase II–III studies; (2) randomised controlled trials; (3) systematic reviews and meta-analyses; (4) experimental studies investigating the molecular mechanisms of TSLP-mediated inflammation; and (5) international guidelines addressing CRSwNP management.

Studies were excluded if they lacked sufficient methodological detail, were duplicate publications, conference abstracts without full-text availability, or did not contain relevant data on inflammatory endotypes or biologic therapies in CRSwNP.

Initial screening was performed based on titles and abstracts, followed by full-text evaluation to assess relevance to the objectives of the review. Particular attention was given to studies evaluating the clinical efficacy of tezepelumab, including changes in Nasal Polyp Score (NPS), Nasal Congestion Score (NCS), Sino-Nasal Outcome Test-22 (SNOT-22), and safety outcomes. Furthermore, data from the PATHWAY, NAVIGATOR, and WAYPOINT clinical trial programmes were analysed, with particular emphasis on sub-analyses relevant to patients with CRSwNP and concomitant bronchial asthma, given shared inflammatory mechanisms.

Data were synthesised using a qualitative narrative approach. The findings were organised into five thematic domains: (1) the role of TSLP in the pathogenesis of CRSwNP; (2) cellular and molecular mechanisms of action of tezepelumab; (3) clinical efficacy of tezepelumab; (4) safety profile; and (5) its role within current biologic treatment strategies for severe CRSwNP.

## 3. Results

### 3.1. The Pathophysiology of Chronic Rhinosinusitis with Nasal Polyps and the Role of Epithelial Alarms

The current understanding of chronic rhinosinusitis with nasal polyps (CRSwNP) has shifted from a concept of a primarily local inflammatory disorder to a model of epithelial–immune interaction, in which impairment of the epithelial barrier plays a central role [34]. Disruption of the nasal epithelial integrity leads to the activation of intracellular signalling pathways and the release of pro-inflammatory mediators in response to environmental, infectious, and allergenic stimuli [35].

The nasal epithelium is recognised not only as a structural barrier but also as a key immunoregulatory interface that orchestrates and maintains inflammatory responses within the mucosa of the upper respiratory tract [1,2,34].

Within the current paradigm of biologic therapy for CRSwNP, the site of therapeutic intervention is a key determinant of treatment response. Biologic agents can be broadly classified according to their position within the inflammatory cascade, ranging from upstream epithelial alarmins to downstream effector cytokines and immunoglobulin E (IgE) [22]. Agents targeting downstream mediators, such as IL-5 or IgE, demonstrate high specificity for well-defined type 2 inflammatory endotypes. In contrast, inhibition of upstream regulators such as thymic stromal lymphopoietin (TSLP) occurs at the epithelial interface, intercepting the inflammatory cascade before activation of both adaptive (Th2) and innate (ILC2) immune pathways [22,36]. Consequently, upstream blockade has the potential to provide therapeutic benefit across a broader spectrum of inflammatory phenotypes and appears to be less dependent on baseline biomarker profiles than biologics targeting downstream mediators [37].

In response to exogenous stimuli, including viral infections, bacterial superantigens, allergens, and environmental pollutants, epithelial cells release a group of cytokines collectively referred to as epithelial alarmins [1,2,35,38]. These include thymic stromal lymphopoietin (TSLP), interleukin-25 (IL-25), and interleukin-33 (IL-33), which play a central role in the initiation and amplification of type 2 inflammatory responses [23,35].

TSLP is a key epithelial-derived cytokine belonging to the IL-7–like family and serves as a central regulator of type 2 inflammatory responses. It orchestrates downstream Th2 immune cascades through interaction with a heterodimeric receptor complex composed of TSLPR and IL-7Rα [23,24,26,32]. The primary cellular target of TSLP is dendritic cells, whose activation induces expression of the costimulatory molecule OX40L, a critical driver of subsequent Th2 differentiation [26,32].

**Scheme 1:**
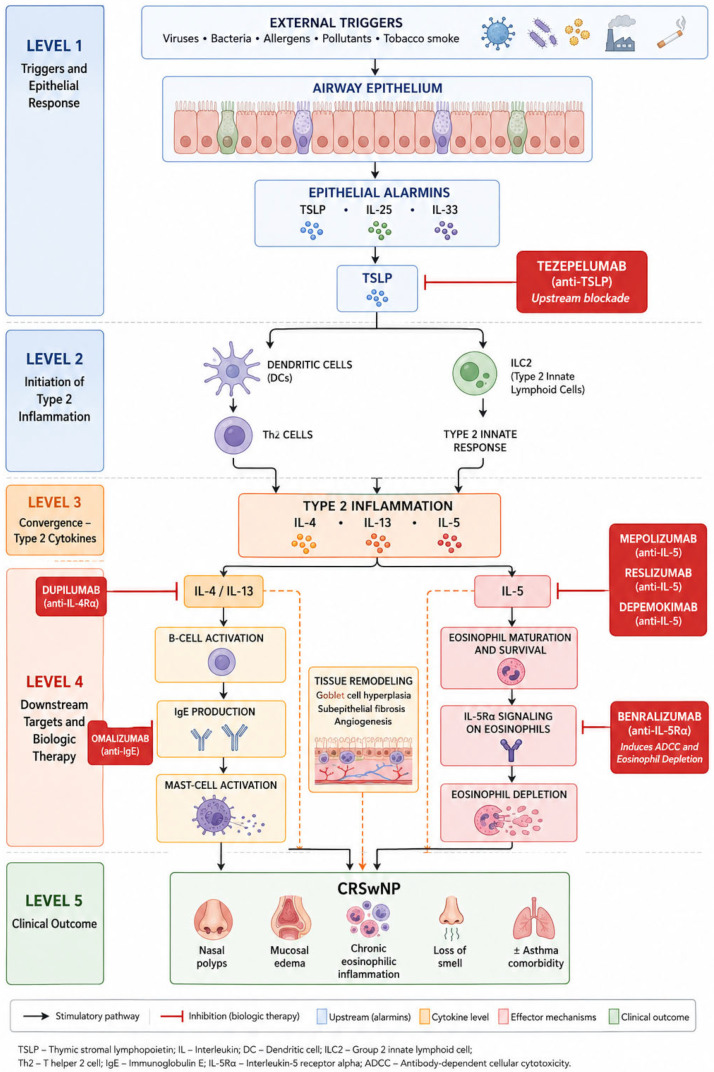
Hierarchical organization of type 2 inflammatory pathways and biologic targets in chronic rhinosinusitis with nasal polyps (CRSwNP). Environmental stimuli trigger epithelial cells to release alarmins, including thymic stromal lymphopoietin (TSLP), which activates dendritic cells and group 2 innate lymphoid cells (ILC2). This initiates the production of the type 2 cytokines IL-4, IL-13, and IL-5, leading to IgE synthesis, mast cell activation, eosinophilic inflammation, tissue remodeling, and ultimately nasal polyp formation. Tezepelumab acts at the most upstream level by inhibiting TSLP. Dupilumab blocks IL-4Rα signaling, omalizumab neutralizes IgE, mepolizumab, reslizumab, and depemokimab target IL-5, whereas benralizumab targets IL-5Rα, resulting in eosinophil depletion.

Activated dendritic cells migrate to regional lymph nodes, where they promote the differentiation of naïve CD4+ T cells into Th2 effector cells [26,32]. This process results in the enhanced production of type 2 cytokines, including interleukin (IL)-4, IL-5, and IL-13, which drive eosinophilic inflammation in CRSwNP [20,28]. The hierarchical organization of these inflammatory pathways and the corresponding molecular targets of biologic therapies are illustrated in Scheme 1.

Beyond the classical Th2-mediated pathway, TSLP also directly activates innate immune responses, particularly type 2 innate lymphoid cells (ILC2) [26,32]. ILC2s produce large amounts of IL-5 and IL-13 independently of antigen presentation, thereby contributing to sustained eosinophilic inflammation. This antigen-independent pathway is considered a key mechanism underlying persistent disease activity and the development of steroid-resistant CRSwNP phenotypes [11,26].

In addition, TSLP regulates multiple effector cell populations within the nasal mucosa. It enhances mast cell activity, leading to increased release of pro-inflammatory mediators even in the absence of IgE-mediated degranulation [26,27]. At the eosinophil level, TSLP promotes prolonged tissue survival and enhanced chemotactic activity, thereby sustaining chronic inflammation [26]. Furthermore, it contributes to B-cell activation, promoting immunoglobulin class switching and increased IgE production, which reinforces the interplay between allergic and non-allergic inflammatory pathways [23,26]. Persistent type 2 inflammation is also associated with tissue remodelling processes, including epithelial–mesenchymal transition [23,34].

TSLP occupies a central position within the immuno-inflammatory cascade in CRSwNP, functioning as an upstream regulator that integrates both innate and adaptive immune responses [23,26]. This upstream localisation makes TSLP a relevant therapeutic target for biologic strategies aimed at early interruption of type 2 inflammatory pathways.

Importantly, the biological functions of TSLP extend beyond the classical type 2 inflammatory pathway [20,30,32]. Increasing evidence indicates that TSLP is involved in broader immunoregulatory networks, including epithelial barrier dysfunction, dendritic cell activation, innate immune responses, and crosstalk with non-type 2 inflammatory pathways [26,31,34]. In addition, TSLP contributes to tissue remodeling, epithelial–mesenchymal transition, and the maintenance of chronic airway inflammation through mechanisms that are not exclusively dependent on eosinophilic responses [23,25,26,34]. These findings support the concept of TSLP as a central upstream regulator of airway inflammation rather than merely a driver of type 2 immunity [20,26,30]. Consequently, TSLP inhibition may provide therapeutic benefit across a broader spectrum of inflammatory endotypes, including mixed and partially non-type 2 phenotypes, which are often associated with greater disease severity and reduced responsiveness to conventional therapies [1,20,26,32].

### 3.2. Tezepelumab: Structure and Mechanism of Action

Tezepelumab is a fully human IgG2λ monoclonal antibody generated using recombinant DNA technology [33]. It binds thymic stromal lymphopoietin (TSLP) with high specificity, thereby preventing activation of upstream inflammatory pathways involved in chronic inflammatory airway diseases [26,33]. Early clinical studies established both the safety of tezepelumab and its capacity to selectively neutralise circulating TSLP in humans [33].

The primary mechanism of action of tezepelumab is the binding and neutralisation of TSLP, thereby preventing its interaction with the heterodimeric TSLPR/IL-7Rα receptor complex expressed on target cells [26,33]. By blocking TSLP signalling, tezepelumab inhibits the activation of key immune cell populations, including dendritic cells, and disrupts the subsequent polarisation of naïve T cells towards a Th2 phenotype. This results in reduced production of the principal type 2 inflammatory cytokines, including IL-4, IL-5, and IL-13 [24,31].

Unlike conventional biologics that target individual downstream cytokines, tezepelumab acts at an upstream level of the inflammatory cascade by blocking TSLP signalling [23,33]. This mechanism allows modulation of multiple components of both innate and adaptive immunity, including pathways involving type 2 innate lymphoid cells (ILC2s), mast cells, eosinophils, and Th2 lymphocytes [26,32].

Recent mechanistic studies further support the concept that TSLP functions as a key upstream regulator linking epithelial injury to immune activation [26,34]. In addition to reducing Th2-associated cytokine production, TSLP inhibition may modulate broader inflammatory networks involved in the pathogenesis of airway disease [20,30,32]. This expanded biological role provides a mechanistic rationale for the use of tezepelumab in patients whose disease extends beyond conventional type 2 inflammatory pathways or is not adequately characterized by established type 2 biomarkers. It may also account for the clinical efficacy of tezepelumab across heterogeneous inflammatory phenotypes observed in recent clinical studies [20,26,33].

An important feature of tezepelumab is that its clinical activity appears to be less dependent on baseline inflammatory biomarkers than that of several currently available biologics [26,33]. Unlike anti-IL-5 and anti-IgE therapies, whose efficacy is often influenced by eosinophil counts or serum IgE levels, TSLP blockade may provide therapeutic benefit across a broader range of inflammatory endotypes, including mixed and partially non-type 2 phenotypes [17,33]. This characteristic may be particularly relevant for patients with severe, recurrent, or steroid-refractory CRSwNP.

Tezepelumab also exerts effects on multiple cellular components of the nasal mucosa. TSLP blockade has been shown to reduce dendritic cell activation, decrease eosinophil survival in tissues, and attenuate mast cell activity, thereby indirectly modulating IgE-mediated inflammatory responses through suppression of Th2 polarisation [26,32,33]. Collectively, these effects contribute to the modulation of the inflammatory microenvironment within the upper airway mucosa.

Consequently, tezepelumab represents the first biologic agent to employ an upstream blockade strategy targeting epithelial alarmins, thereby expanding therapeutic options for chronic rhinosinusitis with nasal polyps, particularly in patients with inadequate responses to currently available targeted biologics.

### 3.3. Clinical Efficacy and Outcomes of Tezepelumab in Chronic Rhinosinusitis with Nasal Polyps

Clinical evidence supporting the use of tezepelumab in chronic rhinosinusitis with nasal polyps (CRSwNP) is rapidly emerging. Current evidence is derived from large randomised clinical trials, including the PATHWAY and NAVIGATOR studies in severe asthma and the Phase III WAYPOINT trial in patients with severe CRSwNP [26,33]. The clinical efficacy of tezepelumab in severe uncontrolled asthma has been established in the PATHWAY and NAVIGATOR studies [33], whereas the WAYPOINT trial provides the first dedicated Phase III evidence supporting its use in severe CRSwNP [26]. Given the substantial overlap in the underlying type 2 inflammatory mechanisms linking CRSwNP and asthma, findings from asthma studies provide important mechanistic context and complementary evidence for the therapeutic potential of tezepelumab in patients with CRSwNP.

One of the principal efficacy endpoints was the change in the Nasal Polyp Score (NPS), an endoscopic measure of nasal polyp burden. Treatment with tezepelumab produced statistically and clinically significant reductions in NPS, with treatment effects becoming evident as early as week 4 and sustained throughout the 52-week treatment period [26]. At week 52, patients receiving tezepelumab achieved a least-squares mean reduction in NPS of 2.47 points from baseline, compared with 0.47 points in the placebo group, corresponding to a between-group least-squares mean difference of −2.01 points (95% CI, −2.33 to −1.68; *p* < 0.001) [26].

Nasal congestion, assessed using the Nasal Congestion Score (NCS), represents one of the principal patient-reported outcome measures in CRSwNP. In the WAYPOINT trial, treatment with tezepelumab was associated with a sustained improvement in nasal congestion compared with placebo, with clinically meaningful differences emerging early during treatment and maintained throughout the 52-week study period [26].

Improvement in olfactory function was another clinically important finding, as loss of smell is among the most disabling and treatment-resistant symptoms of CRSwNP. Patients receiving tezepelumab demonstrated significant improvements in olfactory function, as assessed by the University of Pennsylvania Smell Identification Test (UPSIT), indicating recovery of smell perception during treatment [8,26].

Furthermore, clinically meaningful improvements were observed in the Sino-Nasal Outcome Test-22 (SNOT-22) quality of life score. The reduction in total score exceeded the threshold for minimal clinically important difference (approximately 8.9 points), with mean reductions of more than 24 points, reflecting improvements in both nasal symptoms and overall health-related quality of life [4,26].

A relevant clinical effect of this therapy is its steroid-sparing capacity. Treatment with tezepelumab was associated with a reduction in the need for systemic glucocorticosteroids, with decreases reported in the range of 65–70% [26]. In addition, a reduced requirement for revision functional endoscopic sinus surgery (FESS) was observed [24,26], along with a lower need for delaying surgical intervention. These findings are clinically relevant given the high recurrence rates following sinus surgery [5,13].

Overall, the available evidence indicates that tezepelumab exerts a broad effect on key domains of CRSwNP, including endoscopic findings, symptom burden, and health-related quality of life, supporting its therapeutic potential in this patient population.

In addition to evidence from randomized clinical trials, early real-world data have begun to clarify the effects of tezepelumab on upper airway disease in patients with severe asthma and coexisting CRSwNP [36]. Observational studies have demonstrated improvements in sinonasal symptoms, nasal obstruction, olfactory function, and health-related quality of life following the initiation of tezepelumab therapy [36]. These findings are clinically relevant because asthma and CRSwNP share common epithelial–immune mechanisms that drive chronic airway inflammation [39,40]. Although the available evidence remains limited and is derived predominantly from patients with severe asthma and concomitant CRSwNP, the observed clinical benefits support the potential role of TSLP inhibition in improving manifestations of both upper and lower airway disease [40,41]. Nevertheless, prospective studies specifically designed for CRSwNP populations are required to validate these preliminary observations and better define the therapeutic role of tezepelumab in routine clinical practice [36].

### 3.4. Safety and Tolerability of Tezepelumab

The safety profile of tezepelumab has been evaluated in large clinical programmes, including long-term extension studies with follow-up of up to 104 weeks [26,33]. Comprehensive assessments in patients with severe respiratory diseases have demonstrated consistent tolerability over extended treatment periods [26].

Overall, tezepelumab has shown a safety profile comparable to placebo, with no major safety signals leading to treatment discontinuation in the majority of patients [26,33].

The most commonly reported adverse events are injection site reactions, including erythema, localised swelling, and mild pain, occurring in approximately 5–7% of patients [26]. These events are generally mild and self-limiting and do not require specific intervention.

Reported adverse events include rhinopharyngitis and mild upper respiratory tract infections, with incidence rates of approximately 4–6%, as well as headache occurring in 3–5% of patients [26]. In most cases, these events were transient and did not lead to treatment discontinuation.

From an immunological perspective, tezepelumab does not directly suppress key components of systemic host defence mechanisms. Unlike biologics targeting downstream effector cytokines, TSLP blockade has not been associated with a clinically meaningful increase in serious infections, opportunistic infections, or parasitic infestations [26,33].

Anaphylaxis is rare, occurring in less than 0.1% of cases [26].

Overall, the available data indicate that tezepelumab has a favourable safety and tolerability profile in long-term use.

### 3.5. Comparative Analysis of Biologic Therapies in CRSwNP and the Emerging Role of Tezepelumab (Table 1)

Current biologic therapy for chronic rhinosinusitis with nasal polyps (CRSwNP) includes monoclonal antibodies targeting key pathways of type 2 inflammation. These comprise dupilumab (IL-4Rα inhibitor), mepolizumab and reslizumab (anti-IL-5), and omalizumab (anti-IgE). The clinical positioning and expert recommendations regarding the use of these agents in European practice have been well described in the literature [1,15,42]. Despite their established efficacy, treatment response is largely influenced by the underlying inflammatory endotype, which limits their applicability in patients with heterogeneous or mixed disease patterns [16,17].

Dupilumab acts by blocking IL-4 and IL-13 signalling pathways, resulting in a reduction in nasal polyp burden, decreased need for surgical intervention, and improvement in symptoms, including restoration of olfactory function [15,21]. However, its efficacy may be less pronounced in non-type 2 or mixed inflammatory endotypes [17].

Anti-IL-5 therapies, including mepolizumab and reslizumab, benralizumab are primarily directed at the control of eosinophilic inflammation [15,21]. Their efficacy is supported by reductions in both tissue and peripheral eosinophil counts. However, clinical responses may be attenuated in patients with low baseline eosinophilia or pronounced inflammatory heterogeneity [15,17].

Omalizumab targets IgE-mediated pathways and demonstrates the greatest efficacy in patients with concomitant atopy and an allergic disease phenotype [17,42]. However, its effects on structural remodelling of the nasal mucosa in CRSwNP are variable. Studies exploring localized (intranasal) administration of monoclonal antibodies targeting IL-5 have further highlighted the importance of tissue-specific inflammatory patterns [15].

In contrast, tezepelumab adopts an upstream inhibitory strategy by targeting the epithelial alarmin TSLP [23,26], positioned at an earlier level of the inflammatory cascade. This mechanism enables modulation of both innate and adaptive immune responses, including dendritic cell activation, Th2 differentiation, and ILC2-mediated pathways [26,32].

A key distinguishing feature of tezepelumab is its potential efficacy, which appears to be independent of the underlying inflammatory endotype [26,33]. In contrast to biologics targeting individual cytokines, TSLP blockade may act across a broader spectrum of inflammatory patterns, including mixed and partially non-type 2 phenotypes. These endotypes are typically associated with more severe disease and reduced responsiveness to corticosteroid therapy [23,26].

**Table 1:** Comparative analysis of biologic therapies for chronic rhinosinusitis with nasal polyps (CRSwNP).

Evaluation Criterion	Tezepelumab	Dupilumab	Mepolizumab/Reslizumab	Omalizumab	Benralizumab
Mechanism of action and molecular target	Anti-TSLP monoclonal antibody (Upstream epithelial alarmin inhibition) [23,26]	Anti-IL-4Rα monoclonal antibody (Downstream IL-4/IL-13 signaling blockade) [15]	Anti-IL-5 monoclonal antibodies (Downstream eosinophilic cytokine inhibition) [15,21]	Anti-IgE monoclonal antibody (Downstream free IgE sequestration) [42]	Anti-IL-5Rα monoclonal antibody (Downstream profound eosinophil depletion via ADCC) [18,22]
Pivotal clinical trials	WAYPOINT (2025) [26], NAVIGATOR [33] (asthma trial with CRSwNP subgroup)	SINUS-24, SINUS-52 [15,21]	SYNAPSE (mepolizumab) [15]	POLYP 1, POLYP 2 [42]	OSTRO, ORCHESTRA [18]
Efficacy on Nasal Polyp Score (NPS)	High: Baseline reduction by 1.8–2.2 points at week 52 (WAYPOINT data) [26]	High: Statistically significant decrease in polyp burden and volume [15,21]	Moderate to High: Optimal response observed in severe eosinophilic disease [15]	Moderate: Clinical response predominantly observed in comorbid atopic patients [42]	High: Significant reduction in NPS compared to placebo at week 40 [18]
Dependence on baseline biomarkers	Low: Therapeutic efficacy is independent of T2-high status or baseline eosinophil/FeNO levels [26,32,33]	Moderate: Requires verification of an active Type 2 inflammatory profile [15]	Very High: Clinical response directly correlates with tissue and peripheral eosinophil counts [15,21]	Very High: Clinical efficacy is contingent upon systemic atopic status and elevated total IgE [42]	Very High: Superior efficacy observed in patients with high baseline blood eosinophil counts [18]
Clinical outcomes (Outcome measures)	Significant improvement in sinonasal symptoms (SNOT-22, NPS, TSS), reduction in OCS use [26,32,33]	Significant improvement in all key outcomes (NPS, TSS, SNOT-22, UPSIT), reduction in exacerbation rate and OCS use [15,21]	Reduction in polyp volume and exacerbation rate, symptom (VAS) improvement, OCS sparing effect [15,21,42]	Improvement in symptoms (SNOT-22, TSS), OCS sparing effect, but less impact on polyp size [42]	Improvement in symptoms (TSS, SNOT-22, UPSIT) and quality of life, OCS sparing effect [18,22]
Safety and tolerability	Good safety profile. Main AEs: nasopharyngitis, headache [26,33]	Good safety profile. Main AEs: injection-site reactions, conjunctivitis, arthralgia [15,21]	Good safety profile. Main AEs: injection-site reactions, headache, pharyngitis [15,21,42]	Good safety profile. Main AEs: injection-site reactions, headache, sinusitis [42]	Good safety profile. Main AEs: pharyngitis, headache, injection-site reactions [18,22]
Quality of Life (QoL)	Significant improvement in SNOT-22 (extrapolated from asthma data/confirmed by WAYPOINT) [26,32]	Significant improvement in generic and disease-specific QoL (SNOT-22, EQ-5D) [15,21]	Significant improvement in SNOT-22 quality of life scores [15,42]	QoL improvement, more pronounced in patients with comorbid asthma [42]	Significant improvement in SNOT-22 quality of life scores [18,22]
Reduction in need for surgery	Confirmatory data on reduction in polyp surgery rate expected from WAYPOINT [26]	Clinically significant reduction in the proportion of patients requiring surgery vs. placebo [15,21]	Significant reduction in the proportion of patients requiring surgery (57% reduction in SYNAPSE) [15,21]	Data limited, but studies show a trend towards reduced need for surgery [42]	Reduction in need for surgery compared to placebo (significant in OSTRO) [18,22]

Tezepelumab may be considered a member of a novel class of biologic therapies targeting the early stages of the inflammatory cascade. Its clinical role includes patients with inadequate response to existing interleukin-targeted therapies, recurrent chronic rhinosinusitis, and severe comorbid asthma [15,26].

Given the current level of evidence, direct head-to-head comparisons between tezepelumab and other biologics in CRSwNP remain limited, underscoring the need for further randomised controlled trials to better define its position within treatment algorithms [41].

## 4. Discussion

Chronic rhinosinusitis with nasal polyps (CRSwNP) is a heterogeneous inflammatory disease driven predominantly by type 2 immune responses, although clinically relevant mixed and non-type 2 endotypes contribute to treatment resistance in a subset of patients [1,17,20]. The fact that biological processes vary significantly from patient to patient means that currently available biologics that target downstream mediators such as IL-4/IL-5/IgE are not as effective, particularly in patients with complex or overlapping inflammatory patterns [15,17].

Tezepelumab represents a distinct therapeutic strategy by targeting thymic stromal lymphopoietin (TSLP), an epithelial-derived alarmin located at the apex of the inflammatory cascade [23,26]. By blocking TSLP signalling, upstream activation of dendritic cells, Th2 polarization, and type 2 innate lymphoid cell (ILC2) responses can be reduced, resulting in broader immunomodulation compared with single-cytokine inhibition strategies [26,32]. This upstream positioning has the potential to allow activity across a range of inflammatory endotypes, including those that are less responsive to conventional biologics [17,26].

Recent advances in the understanding of TSLP biology indicate that its function extends beyond canonical type 2 inflammation [20,26,32]. TSLP is increasingly recognized as a central regulator of epithelial–immune interactions, influencing both innate and adaptive immune responses while contributing to airway remodeling and persistent mucosal inflammation [20,26,30]. This expanded biological role may partly explain the therapeutic effects of TSLP inhibition in patients whose disease is not fully characterized by elevated type 2 biomarkers [1,20,26,33]. Consequently, tezepelumab may represent a broader therapeutic strategy than conventional biologics targeting individual downstream type 2 mediators, with potential applicability across a wider range of inflammatory endotypes [20,26,32].

Clinical evidence from the WAYPOINT trial and supporting clinical programmes indicates that tezepelumab may reduce nasal polyp burden, alleviate symptoms, and reduce systemic corticosteroid requirements, while enhancing patient-reported outcomes [26]. However, a significant proportion of the mechanistic and clinical rationale is still derived from asthma populations, including the PATHWAY and NAVIGATOR trials [33], and only partially validated in CRSwNP-specific cohorts [26]. Therefore, while it is biologically plausible to extrapolate the efficacy of one airway disease to another, due to the presence of shared epithelial–immune pathways, this should be interpreted with caution [20,32].

Furthermore, emerging real-world evidence from patients with severe asthma and coexisting CRSwNP further supports the clinical relevance of TSLP blockade in upper airway disease [39]. Improvements in sinonasal symptoms, olfactory function, and patient-reported outcomes observed in routine clinical practice suggest that the therapeutic benefits of tezepelumab extend beyond the controlled setting of randomized clinical trials [39,40]. Although these findings remain preliminary and are derived predominantly from asthma populations with coexisting CRSwNP, they reinforce the concept of upstream epithelial alarmin inhibition as a promising therapeutic strategy for united airway disease. Nevertheless, dedicated prospective studies in patients with CRSwNP are required to confirm these observations and establish the long-term effectiveness of tezepelumab in routine clinical practice [39,40].

It is important to note that there are currently no head-to-head comparative trials available between tezepelumab and established biologics in CRSwNP [15]. As a result, the precise positioning of this technology within existing treatment algorithms is not yet clear. Furthermore, the absence of defined predictive biomarkers of response has hindered the precision stratification of patients. Recent reviews have highlighted the need for head-to-head comparative trials and validated predictive biomarkers to optimize patient selection among the expanding range of available biologic therapies [22,37]. Long-term data on disease modification, including effects on epithelial remodelling and postoperative recurrence, also remain insufficient [17,22,26,36].

From a clinical perspective, tezepelumab may be particularly relevant for patients with severe, recurrent CRSwNP with inadequate response to existing biologics or those with mixed inflammatory endotypes [15,26]. However, its definitive therapeutic niche requires validation through dedicated randomised controlled trials directly enrolling CRSwNP populations.

Tezepelumab is a promising biologic that targets epithelial alarmin-mediated inflammation. This indicates a shift towards earlier intervention in airway disease pathophysiology; however, current evidence is insufficient to establish superiority, optimal patient selection, or long-term clinical benefit [26,33].

## 5. Conclusions

Tezepelumab is a first-in-class biologic targeting thymic stromal lymphopoietin (TSLP), an upstream epithelial alarmin involved in the initiation and amplification of type 2 inflammatory responses in chronic rhinosinusitis with nasal polyps (CRSwNP) [23,26]. By acting at an early level of the inflammatory cascade, it provides a mechanistically broader immunomodulatory effect compared with currently available biologics directed against downstream cytokines [26,32].

Available clinical evidence suggests that tezepelumab may improve key disease outcomes, including nasal polyp burden, nasal obstruction, olfactory function, and quality of life, while also reducing systemic corticosteroid use [26]. These findings support its potential role in patients with severe, recurrent, or treatment-refractory CRSwNP.

However, the current evidence base remains limited and is largely derived from studies in asthma populations, with only emerging CRSwNP-specific data. In addition, the absence of head-to-head comparisons with established biologics and a lack of validated predictive biomarkers restrict its precise positioning in current treatment algorithms [15,26].

Further randomized controlled trials directly in CRSwNP populations are required to confirm long-term efficacy, safety, and disease-modifying potential, as well as to define the optimal patient phenotype for therapy selection.

Overall, tezepelumab represents a promising upstream therapeutic approach that expands the biological treatment landscape in CRSwNP and supports a shift toward earlier intervention in epithelial–immune-driven airway inflammation.

## Data Availability

No new data were created or analyzed in this study. Data sharing is not applicable to this article.

## Abbreviations

The following abbreviations are used in this manuscript:

CRSwNP	Chronic rhinosinusitis with nasal polyps
FESS	functional endoscopic sinus surgery
TSLP	thymic stromal lymphopoietin
ILC2	type 2 innate lymphoid cell
